# Supplementary material for: Rapid turnover of life-cycle-related genes in the brown algae
Source: Genome Biol. 2019 Feb 14;20:35. doi: 10.1186/s13059-019-1630-6 (PMC6374913; doi:10.1186/s13059-019-1630-6)
Supplement: Supplementary file 3 — Protein alignments, corresponding Gblocks html file and CDS sequences for the lineage conserved gametophyte-biased genes. (ZIP 2109 kb) [file 13059_2019_1630_MOESM3_ESM.zip › Con_GA_alignments/Ec-08_000720.aa_ali.fasta-gb.htm]

Ec-08\_000720.aa.fasta.trans.aa\_ali.fasta


## Gblocks 0.91b Results

Processed file: **Ec-08\_000720.aa.fasta.trans.aa\_ali.fasta**  
Number of sequences: **2**  
Alignment assumed to be: **Protein**  
New number of positions: **415** (selected positions are underlined in blue)

```
                         10        20        30        40        50        60
                 =========+=========+=========+=========+=========+=========+
Ec-08_000720     --MVHISEDLTTGTKR---SRSARRSRSGAKRVTEAGYPTGVSGGEYKMVNGLRLATLLF
MSTRG.49349.1    HRFVAAQRAWSAGAVPRTYPKQSNRTVTTLHSVLHHSFHRAQQRYQSQDMAIITIRTWAT
                    ##########                                           ####


                         70        80        90       100       110       120
                 =========+=========+=========+=========+=========+=========+
Ec-08_000720     VRVALVALSNARGTAVVEAAPGAPLGLYYERWQQKQTEQTKLPLLLGFTSPINGTRVEGP
MSTRG.49349.1    ALCAWAAISSHTRTHVAGATR-LLRGQHEQEHRQLDGQTEKLPFLLAFTNPMNGSRVEGP
                 ###################      ###################################


                        130       140       150       160       170       180
                 =========+=========+=========+=========+=========+=========+
Ec-08_000720     NVFLHYKIFNTL--ENRELTSAEVRDLSVSTTVCFKLHGFNNVPEICAPLRVTTVTIKDA
MSTRG.49349.1    DVSLYYKIFSTAGGGGRELTSSEIQRLSRTTTVCFELRGFDNAPEICAPLRVSAVTIKDA
                 ###########     ############################################


                        190       200       210       220       230       240
                 =========+=========+=========+=========+=========+=========+
Ec-08_000720     LPAKWHHVTGSIKDTRTGKLLGASDGGVSVFNNLNGYGVLDMCGESACLDSNALRSAYFD
MSTRG.49349.1    LPAKWHHVTGRMMDSITDELVGSSHDGIYVFNSLNGYGVLDMCGETACLDSSDLRAAYFD
                 ############################################################


                        250       260       270       280       290       300
                 =========+=========+=========+=========+=========+=========+
Ec-08_000720     YVYRHPSWYGRIPLTG--ASKLSTTFSIRSGLSNVISAFNIKSMLDAPCGDLSWMPLVSG
MSTRG.49349.1    YIYRHPRWNGHPPSSTGIGSKISSTFSIRAGLAGVLSAFNIKSMLDAPCGDLTWMPLVTG
                 #############      #########################################


                        310       320       330       340       350       360
                 =========+=========+=========+=========+=========+=========+
Ec-08_000720     IQNVQYTGADIVEIAVETNRKKFG--ADITEIGDEDLEVGREIAAAMRRGEGLKDPAFVV
MSTRG.49349.1    IQDVAYTGADIVDIVVESNREKFGPGRAIGDEVVEAAELGEHVAAVMRRGEGLRDPVFVT
                 ########################    ################################


                        370       380       390       400       410       420
                 =========+=========+=========+=========+=========+=========+
Ec-08_000720     ADLVEGVPASQDGAPFDLIFVRDLMVHLPARHNLKVIQNIQASGARFLMASTYVEADENK
MSTRG.49349.1    ADLVEGVPASQDGKPFDLVFVRDLMVHLPARHNLKVIQNIQSSGARFLMASTYVEADENE
                 ############################################################


                        430       440       450       460       470       480
                 =========+=========+=========+=========+=========+=========+
Ec-08_000720     LSETFVPPIGHNINLSLPPYCLPEPMAVFRDDSTERTDLRMGLWDLHEHSPLALSGDCSP
MSTRG.49349.1    VSESFVPPVGHNINLSLPPYCLPEPIALIRDDSTDRTDRRMGLWDLHQDSPLALSGDCLA
                 ############################################################


                 
                 =====
Ec-08_000720     TSVAV
MSTRG.49349.1    MSTTA
                 ##
```

```
Parameters used
Minimum Number Of Sequences For A Conserved Position: 2
Minimum Number Of Sequences For A Flanking Position: 2
Maximum Number Of Contiguous Nonconserved Positions: 8
Minimum Length Of A Block: 10
Allowed Gap Positions: None
Use Similarity Matrices: Yes
```

```
Flank positions of the 6 selected block(s)
Flanks: [4  13]  [57  79]  [86  131]  [137  253]  [260  324]  [329  482]  

New number of positions in Ec-08_000720.aa.fasta.trans.aa_ali.fasta-gb:  415  (85% of the original 485 positions)
```
